# The Role of TIM-3 in Hepatocellular Carcinoma: A Promising Target for Immunotherapy?

**DOI:** 10.3389/fonc.2020.601661

**Published:** 2020-12-03

**Authors:** Mazdak Ganjalikhani Hakemi, Morteza Jafarinia, Mahdieh Azizi, Mahsa Rezaeepoor, Orkhan Isayev, Alexandr V. Bazhin

**Affiliations:** ^1^Department of Immunology, School of Medicine, Isfahan University of Medical Sciences, Isfahan, Iran; ^2^Department of Immunology, School of Medicine, Hamedan University of Medical Sciences, Hamedan, Iran; ^3^Department of Cytology, Embryology and Histology, Azerbaijan Medical University, Baku, Azerbaijan; ^4^Genetic Resources Institute, Azerbaijan National Academy of Scince, Baku, Azerbaijan; ^5^Department of General, Visceral and Transplant Surgery, Ludwig-Maximilians University of Munich, Munich, Germany; ^6^German Cancer Consortium (DKTK), Partner Site Munich, Munich, Germany

**Keywords:** hepatocellular carcinoma, T cell immunoglobulin mucin-3, cancer, immune cells, immunometabolics

## Abstract

One of the most common tumors in the world is hepatocellular carcinoma (HCC), and its mortality rates are still on the rise, so addressing it is considered an important challenge for universal health. Despite the various treatments that have been developed over the past decades, the prognosis for advanced liver cancer is still poor. Recently, tumor immunotherapy has opened new opportunities for suppression of tumor progression, recurrence, and metastasis. Besides this, investigation into this malignancy due to high immune checkpoint expression and the change of immunometabolic programming in immune cells and tumor cells is highly considered. Because anti-cytotoxic T lymphocyte–associated protein (CTLA)-4 antibodies and anti-programmed cell death protein (PD)-1 antibodies have shown therapeutic effects in various cancers, studies have shown that T cell immunoglobulin mucin-3 (TIM-3), a new immune checkpoint molecule, plays an important role in the development of HCC. In this review, we summarize the recent findings on signal transduction events of TIM-3, its role as a checkpoint target for HCC therapy, and the immunometabolic situation in the progression of HCC.

## Introduction

Primary liver cancer is the sixth most frequent type of cancer and the second or third most common cause of cancer-related death globally with about 782,500 deaths per year. The term “hepatobiliary cancer” refers to primary malignancies in the liver, either hepatocellular carcinoma (HCC) or the intra- and extra-hepatic biliary ductal system, giving rise to biliary tract carcinomas (BTC). HCC is the predominant type of primary liver cancer, which comprises approximately 90% of cases, whereas BTCs are rare tumors (1–3). Surgery plays a substantial role in the cure in the early stages of the disease and is restricted to a minority of patients with localized diseases; however, most HCC patients are diagnosed at advanced stages when therapeutic options are limited, and apart from locoregional or systemic therapies, recurrence happens in most of them within 5 years. Currently, sorafenib and lenvatinib, which are oral tyrosine kinase inhibitors, as the ﬁrst-line treatment have shown efficacy in late stages of HCC and provide a short increase of median overall survival for patients. Also, other angiogenesis inhibitors, such as cabozantinib; regorafenib; and ramucirumab, a monoclonal antibody directed against the vascular endothelial growth factor receptor (VEGFR2), are second-line therapeutic options (1, 4).

Despite the various treatments that have been developed over the past decades, the prognosis for advanced liver cancer is still poor. Therefore, new approaches are needed to treat liver cancer (5, 6). In recent years, tumor immunotherapy has been proposed as a promising method for suppression of tumor progression, recurrence, and metastasis (3). In particular, most recent studies have focused on the role of the immune system in HCC (1). “Immunometabolism” is a term that has been recently proposed, and it points to the functional intracellular metabolic changes that occur within different immune cells. Understanding the immunometabolic situation of T lymphocytes and other immune cells, along with the metabolic behavior of cancer cells, can help us to design new therapeutic approaches against cancers (3, 5). On the other hand, the role of the immune system and immune checkpoint inhibitors in HCC is taken into consideration in recent studies, and they have shown significant progress in the last few years (1, 2). Accordingly, one of the main strategies to improve the specific immune response to the tumor is to use monoclonal antibodies to block the immune checkpoint (1, 3). The most recognized immune checkpoint proteins include cytotoxic T lymphocyte–associated protein 4 (CTLA-4), programmed cell death protein 1 (PD-1), PD-L1, V-domain Ig suppressor of T cell activation (VISTA), T cell immunoglobulin and mucin domain-containing protein 3 (TIM-3), lymphocyte activating 3 (LAG-3), and OX40. Among these, CTLA-4 and PD-1 inhibitors have been well characterized and are approved by the FDA for treating melanomas with some progress for application in treating HCC (3). Apart from important results that have already been obtained by some studies on immunotherapy for HCC, future studies must present more specific immune targets and novel immune checkpoints (3). TIM-3 is a less investigated immune checkpoint molecule that plays important and complex roles in regulating immune responses and in inducing immune tolerance (7). Therefore, investigating the expression pattern and the detailed role of TIM-3 in the metabolic alteration of tumor-infiltrating lymphocytes (TILs) as well as the tumor cells of HCC is of critical importance in finding more specific and effective therapeutic approaches in the future. Here, we review the cutting-edge findings of signal transduction events in TIM-3, its role as a checkpoint target for HCC therapy, and its immunometabolic situation in the progression of HCC. This would help in understanding whether the TIM-3 immunometabolic checkpoint molecule could be recognized as a potential target for HCC therapy or not.

## The Structure of TIM-3 as a Checkpoint Molecule

Innate and adaptive immune cell activation is highly regulated by a series of surface receptor–ligand pairs or immune checkpoints that affect signaling events. Because these molecules have a key role in T cell activation, tolerance, and exhaustion, nowadays, their therapeutic modulation provides effective new treatment strategies in infectious diseases, autoimmunity, allogeneic transplantation, and cancer (8). Although substantial clinical responses have been achieved in cancer patients treated with antibodies against the CTLA-4 and PD-1/PD-L1 checkpoints, only a small portion of patients have responded to such therapies in several cancers. Thus, exploring novel co-inhibitory molecules and immune checkpoints for cancer treatment is urgently required, and TIM-3 is a potential candidate (9–11).

The TIM gene family, which includes 8 members (TIM-1 to -8) in mice on chromosome 11B1.1 and 3 members (TIM-1, TIM-3, and TIM-4) in humans on chromosome 5q33.2, was first discovered in 2001 and identified as immune checkpoints that play a vital role in immunoregulation. These molecules are normally expressed as type I surface glycoproteins containing an amino-terminal immunoglobulin variable domain (V domain) with five noncanonical cysteines, a mucin stalk, a transmembrane domain, and a cytoplasmic tail. Among this family, TIM-3, encoded by the HAVCR2 gene and its ligands, is presently attracting increasing attention because of its potential as a target for immunotherapy of different diseases, such as cancers (9, 11, 12).

## Ligands of TIM-3

**Figure 1:**
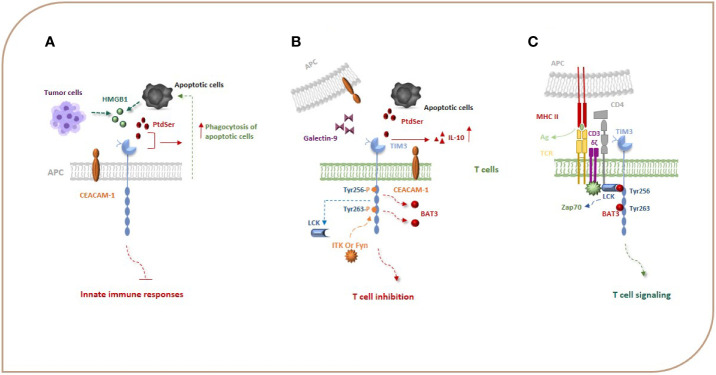
TIM-3, its ligands, and signal transduction events. Gal-9, PtdSer, CEACAM1, and HMGB-1 are the most important ligands for TIM-3. HMGB1 can bind to TIM-3 expressed on APCs and suppress innate immune responses. PtdSer and TIM-3 interaction leads to an increase in the phagocytosis of apoptotic cells **(A)**. Binding of TIM-3 to Gal-9 and other TIM-3 ligands leads to phosphorylation of Y256 and Y263 by the tyrosine kinase ITK or Fyn and release of Bat3 from the TIM-3 tail, which promotes TIM-3-mediated T cell inhibition **(B)**. When TIM-3 is not bound by a ligand, Bat3 links to the C-terminal tail of TIM-3 (the Tyr 256 and Tyr 263 in the C-terminal tail) and blocks SH2 domain-binding sites in its tail and recruits the active catalytic form of LCK that promotes T cell signaling **(C)**. Bat3, HLA-B associated transcript 3; CEACAM1, carcinoembryonic antigen cell adhesion molecule 1; Gal-9, galectin-9; HMGB-1, high-mobility group protein 1.

TIM-3 as a co-inhibitory molecule plays important and complex roles in regulating immune responses and in inducing immune tolerance (7, 13). The extracellular part of TIM-3 includes a mucin domain and an IgV domain, which have glycosylated sites and bind to ligands of TIM-3 (12, 14, 15). Emerging evidence has shown that TIM-3 has several ligands, including C-type lectin galectin-9 (Gal-9), phosphatidylserine (PtdSer), carcinoembryonic antigen cell adhesion molecule 1 (CEACAM1), and high-mobility group protein 1 (HMGB-1) (**Figure 1**). Gal-9 was identified as the first TIM-3 ligand that is highly expressed in immune system tissues, such as lymph nodes, bone marrow, thymus, and spleen, and can also be secreted by tumor cells, endothelial cells, and other cells and interacts with oligosaccharides on the TIM-3 IgV domain (7, 14, 16). The TIM-3–Gal-9 interaction plays significant roles in the consequences of infection, inflammation, peripheral tolerance, tumor immunity, and autoimmunity, and evidence shows that this interaction suppresses the response of immune cells (14, 17). Also, TIM-3 signaling induced by Gal-9 ligation causes exhaustion and apoptosis of antigen-specific Th1 cells and CTLs through the calcium-calpain-caspase-1 pathway, which correlates with the impaired antitumor immune response (7, 13, 18). Collectively, data show that the TIM-3–Gal-9 interaction can act *via* different mechanisms and on different cell types to suppress immune responses.

The TIM-3 expressed on monocytes, macrophages, and DCs acts as a phagocytic receptor for apoptotic cells and enhances the phagocytosis of apoptotic cells *via* interaction with PtdSer, thereby boosting antigen cross-presentation (**Figure 1A**) (19). The biological relationship between the TIM-3 and PtdSer in T cells is unknown as T cells do not have a role in removing apoptotic bodies, but it seems that the interaction of TIM-3 with PtdSer can be effective in the production of IL-10 by T cells because IL-10 has been demonstrated to be expressed by TIM-3^+^ T cells (**Figure 1B**) (20). The HMGB1 as a damage-associated molecular pattern (DAMP) that binds to DNA released from dying cells boosts nucleic acid sensing by toll-like receptors (TLRs), but during chronic infection and cancers, due to HMGB1 binding to TIM-3 expressed on APCs, it seems that this interaction suppresses innate immune responses to the nucleic acid (**Figure 1A**) (13, 21). CEACAM1 is another newly identified ligand for TIM-3 that is co-expressed with TIM-3 in T cells and also is expressed by DCs and other APCs (**Figure 1B**). Therefore, the TIM-3 and CEACAM1 form an axis that can inhibit immune responses either in cis or trans T cells and myeloid cells, thereby downregulating their antitumor immunity. The stability of mature TIM-3 on the cell surface is promoted *via* cis interaction, and both cis and trans interactions mediate the inhibitory function of TIM-3 (16, 22).

## Signal Transduction Events of TIM-3

In contrast to other co-stimulatory and co-inhibitory molecules on immune cells, TIM-3 does not have a classical immunoreceptor tyrosine-based inhibition (ITIM) or immunoreceptor tyrosine-based switch (ITSM) motif in its cytoplasmic tail; however, it contains five tyrosine residues that are conserved between humans and mice. In humans, phosphorylation of two of the tyrosine residues, Y265 and Y272 (Y256 and Y263 in mice), has been shown to be critically important for triggering downstream signaling pathways. Van de Weyer et al. demonstrated that Y265 of human TIM-3 is specifically phosphorylated by the enzyme interleukin inducible T cell kinase (ITK) from the TEC family of kinases when TIM-3 has an interaction with Gal-9 (23). Another study using the Jurkat, D10, and HEK293T cell lines, has shown that the Src family of kinases, Fyn and Lck, can phosphorylate a TIM-3 cytoplasmic portion (15, 24). Intriguingly, evidence suggests that TIM-3 may act as a constitutive positive regulator of T cell function and enhance signaling pathways that lead to T cell activation, at least under conditions of acute stimulation. J. Lee et al. reported that the recruitment of SH2 domain-containing proteins, including the p85 of PI3K and PLC-γ1 to the phosphorylated tail of TIM-3 in the Jurkat T cell line stimulated by TCR/CD3 and CD28, enhances the secretion of IL-2 and the induction of transcription factors important for T-cell activation, i.e., NFAT, AP-1, and NF-kB *via* interaction of the components of the TCR signaling pathway, such as Zap-70 and SLP-76 (24). Also, it has been reported that TIM-3 expression has been associated with the generation of short-lived effector CD8^+^ T cells in an mTOR-dependent manner, which was considered a sign of co-stimulation by TIM-3 (16, 25). These observations led to the ongoing debate of whether TIM-3 is a co-stimulatory or co-inhibitory receptor. The results in the following demonstrate that the pro-inﬂammatory proﬁle of T cells expressing ectopic TIM-3 was abrogated in the presence of an agonistic TIM-3 antibody (24). It seems that any co-stimulatory function of TIM-3 could be reversed in the presence of its ligand(s) (25). Finally, studies have illustrated that, when TIM-3 is not bound by a ligand, the HLA-B associated transcript 3 (Bat3) links to the C-terminal tail of TIM-3, blocks SH2 domain-binding sites in its tail, recruits the active catalytic form of LCK that promotes T cell signaling, and represses TIM-3-mediated cell death and exhaustion (**Figure 1C**). On the other hand, Gal-9 and CEACAM-1 binding to TIM-3 leads to phosphorylation of Y256 and Y263 by the tyrosine kinase ITK or Fyn and release of Bat3 from the TIM-3 tail. This, in turn, promotes TIM-3-mediated T cell inhibition by facilitating the binding of Src kinases and subsequent regulation of TCR signaling (**Figure 1B**) (8, 15, 20, 26). Fyn can potentially contribute to TIM-3-mediated inhibitory signaling as it is known to bind to the same domain in the cytoplasmic tail of TIM-3 as Bat3, indicating potential competition between Fyn and Bat3 for TIM-3 binding, a likely molecular switch between TIM-3/Bat3, and TIM-3/Fyn might change the TIM-3 function from being permissive to TCR signaling to suppression of TCR signaling. TIM-3 can also bind to receptor phosphatases CD45 and CD148 and interrupt immunological synapses, which has been suggested as an additional mechanism of T cell inhibition (16). It can be concluded that TIM-3 acts like a checkpoint inhibitor when it is bound to its ligands. Based on the abovementioned studies, TIM-3 transduces co-stimulatory signals when it is not bound to its ligands. For immunotherapy of cancers, we suggest that using an antagonist of TIM-3 ligands may have more beneficial effects than blocking TIM-3. It is an idea that should be studied in the future.

## The Effect of TIM-3 on Adaptive and Innate Immune Cells

It has been shown that the expressed TIM-3 on immune cells can regulate both innate and adaptive immune responses *via* either positive or negative effects depending on the cells expressing this receptor, its connection to its ligands, and also its microenvironment (27). TIM-3 was initially identified as a T cell marker for Th1 and CD8^+^ T cells in the EAE model (28), and it was later shown that TIM-3 is expressed by human Th1 and Th17 cells (29) and also other cells including DCs, macrophages, microglial cells, mast cells, and NK cells.

## The Effect of TIM-3 on Adaptive Immune Cells

Signaling *via* TIM-3 after binding to the ligand plays a key role in the regulation of immune responses *via* negatively regulating Th1 cell viability and secretion of cytokines, such as TNF and IFN-γ, and this, in turn, leads to immune tolerance (20, 30). It has been reported that TIM-3 also has a role in regulating Th17 cells and suppresses their activation and cytokine secretion (9, 20). However, altered TIM-3 expression was reported to occur in various human diseases. For instance, TIM-3 has been recognized as a negative regulator of antitumor immunity. The expression of TIM-3 on both CD4^+^ and CD8^+^ TILs could induce T cell exhaustion and promote the expansion of the immunosuppressive cell population (12). Recent studies have shown that TIM-3 expressed on T cells and cancer stem cells also suppresses immune responses through inducing the development of myeloid-derived suppressor cells (MDSCs) that are a heterogeneous CD11b^+^Gr-1^+^ myeloid cell population. Blocking of TIM-3 has been shown to reduce the number of MDSCs; increase the production of IL-6, IFN-γ, and TNF-α; and restore effector functions of exhausted CD8^+^ T cells and their antitumor activity (13, 19, 31). Furthermore, TIM-3 is expressed on Foxp3^+^ Tregs, and the interaction of Gal-9 with TIM-3^+^ in these cells enhances the regulatory function of Tregs, such as the robust expression of IL-10, perforin, granzyme A, granzyme G, and CD39 (9, 18, 20). On the other hand, Gal-9 expression on Tregs suppresses TIM-3^+^ effector T cell function, so TIM-3 has been identified as a marker of Tregs in tumors (16, 25). Also, with the expression of TIM-3 on Tfh cells, it was reported in the tumor microenvironment that te TIM-3^+^ Tfh cells produce less of the chemokine CXCL13 and cytokine IL-21 and have less proliferating potential than TIM-3^−^ Tfh cells (32). Accordingly, blocking the TIM-3–Gal-9 pathway entails a palpable decrease in the suppressive activity of Tregs *in vitro* (9). It has been demonstrated in some tumors, including ovarian carcinoma, that blockade of TIM-3 has reversed Treg-mediated immune suppression (18).

Expression of TIM-3 on CD8^+^ T cells is closely associated with other immune checkpoint molecules, including PD-1, CD160, 2B4, and lymphocyte-activation gene 3 (LAG3), and their chronic persistence is associated with an exhaustion status. In comparison with PD-1, single positive CD8^+^ T cells, TIM-3^+^ PD-1^+^ CD8^+^ T cells represent an “extremely” exhausted T cell population that exhibits a lower secretion of IFN-γ and the expression of CD107a, and also the expression of MHC-I on these lymphocytes decreased (9, 20, 33). Dolina et al. have reported that TIM-3^+^ CD8^+^ T cells induced in the liver during adenovirus infection exhibit suppressive “Treg” function. On these suppressive CD8^+^ T cells, TIM-3 acts as a “sink” to uptake HMGB1, preventing HMGB1 from activating hepatic CD8^+^ T cells *via* TLRs (20, 34). Based on this, high levels of TIM-3 expression on CD8^+^ T cells have been reported to be associated with a poor prognosis for tumor progression (18). Based on the expression of TIM-3 in TME and its suppressive effect on adaptive immune cells, it can be suggested that blocking PD-1, which is used as an immunotherapy for a few cancers, may not have a sufficient effect in contrast to blocking PD-1 and TIM-3 together (**Table 1**).

**Table 1:** Expression and function of TIM-3 on immune cells.

Cell type	Function	Reference
Th1	Inhibits Th1 function and important for tolerance	(35)
	Tim-3+ Th1 undergo gal-9-induced cell death	(36)
	Represses Th1 responses in EAE	(26)
Th17	Blockade enhances Th17 polarization *in vivo*	(37, 38)
Treg	Tim-3+PD-1+ Treg suppress allograft rejection	(39)
	Tim-3 signaling is essential for Treg-mediated suppression of allograft rejection	(40, 41)
T CD8^+^	Blockade exacerbates CD8+T cell-mediated EAE	(42)
	Tim-3 marks deeply exhausted CD8+ tumor-infiltrating lymphocytes	(43, 44)
Macrophage	Inhibits the release of pro-inflammatory cytokines in DSS colitis	(45)
	Promotes expression of TGF-β, TNF-α, and IL-1β in microglia	(46)
	Promotes phagocytosis by microglia	(46)
	Tim-3 expressed on peripheral blood monocytes and TAMs in patients with HCC. Enhancing TGF-β-mediated alternative activation of macrophages.	(47)
NK	May promote dysfunction in cancer	(48)
	Induces maternal–fetal tolerance	(49, 50)
	Tim-3 expressed on activated CD56+NK cells act an activating coreceptor on NK cells and promotes IFN-γ production	(51, 52)
Mast cell	Mediates mast cell activation following IgE sensitization and antigen-dependent activation	(53)
	Tim-3 blockade promotes IL-4, IL-6, and IL-13 production and suppresses mast cell apoptosis	(54)
DCs	Inhibits the activation of nucleic acid sensing TLRs	(55)
	Reduces CXCL9 secretion by tumor cDC1s and dampens CD8+ T cell cytotoxicity indirectly	(56)

## The Effect of TIM-3 on Innate Immune Cells

As mentioned earlier, TIM-3 is also expressed on innate immune cells, and after the attachment to its ligand, it regulates the activation of these cells through a mechanism usually studied in T cells. Of note, TIM-3 plays a much more complex role in innate immunity than in adaptive immunity. Zhang et al. have reported that, in autoimmune diseases, TIM-3 expression is increased on macrophages. Moreover, it has been shown that M2-like macrophages show higher levels of TIM-3 expression than M1-like macrophages, and this increased expression of TIM-3 on M2 macrophages participates in immune regulation by suppressing macrophage activation (18, 20). In humans, constitutive expression of TIM-3 on monocyte/macrophages inhibits IL-12 secretion. Although studies about how TIM-3 affects monocytes and macrophages are infrequent, it is known that some mechanisms can be regulated by TIM-3 in these cells in different pathologic conditions, such as infections, autoimmunity, and cancers (15). There appears to be a close connection between TIM-3 expression and the modulation of TLR activation in myeloid cells. Monocytes/macrophages have a high expression of TIM-3 with low cytokine production in a quiescent state, but when these cells are stimulated with TLR, the TIM-3 expression is reduced, and subsequently, the IL-12 production is enhanced. In macrophages, Gal-9–TIM-3 interaction downregulates TLR signaling, leading to a decrease in IL-12 and IL-1β production; an increase of IL-23, IL-6, IL-8, and IL-10 production; and reducing phosphorylation of STAT-1 while enhancing activation of STAT-3, so TIM-3 is a negative regulator of the TLR response (9, 15). According to studies, it seems that TIM-3 can also have TLR-independent effects on macrophages. Xingwei Jiang et al. have reported that TIM-3, through residues Y256 and Y263, binds to STAT1 and inhibits STAT1 phosphorylation and nuclear translocation. This leads to the inhibition of the STAT1-miR-155 signaling axis and enhances the function of SOCS1 in macrophages. As a result, this promotes M2 macrophage polarization (57). Furthermore, studies have shown that TIM-3 is involved in M2 polarization in both TGF-β-dependent and -independent manners and promotes tumor growth through the NF-κB/IL-6 axis (20, 47, 58). TIM-3, except for inhibiting the activation of macrophages, can contribute to the efferocytosis process, which refers to the mechanism of eliminating the damaged cells and apoptotic bodies that happen after binding PtdSer to TIM-3 (15, 20). As the interaction of PtdSer with TIM-3 is the principal signal for the phagocytosis of apoptotic cells, blocking this interaction can induce immunological abnormalities, such as generating autoantibodies (15). Conclusively, TIM-3 can simultaneously enforce and regulate multiple important functions in macrophages, such as the endocytosis of apoptotic bodies and activation in response to TLR stimuli and mechanisms after activation.

The constitutive expression of TIM-3 is found on DCs and may be positively correlated with DC activation. In vitro experiments have shown that the stimulation of TIM-3 with Gal-9 on DCs can result in the production of pro-inflammatory cytokines, such as TNF-α and DC maturation. Additionally, TIM-3 signaling activated by Gal-9 in tumor-bearing animal models can increase the number of mature DCs and improve the antitumor immune response. Nevertheless, some researchers have reported opposing results, demonstrating that TIM-3 may also be a negative regulator of DCs. For example, it has been seen that TIM-3 negative bone marrow-derived DCs (BMDCs), in comparison with TIM-3^+^ DCs, show an impaired function phenotype and exhibit a much poorer capacity to express IFN-β1, IFN-α, and IL-6 cytokines (9, 20). Also, in TME, TIM-3 expression is induced on resident DCs so TIM-3^+^ myeloid cells may act as a molecular sink for HGMB1, which can also be secreted by tumor cells (13, 21). When TIM-3 binds to HMGB1, the transport of nucleic acids into endosomes is blocked; thus, the pattern-recognition receptor-mediated innate immune responses against tumor-derived nucleic acids is suppressed (55). Interestingly, tumor-infiltrating DCs in TME express a higher amount of TIM-3 than DCs in normal tissues. Thus, TIM-3 plays a role in tumor pathogenesis. These findings suggest that TIM-3, which acts as a negative regulatory checkpoint, is associated with both T cell exhaustion and inhibition of innate immune responses (20). The regulatory effect of TIM-3 on DCs is alternating, and other factors, such as different ligands of TIM-3, can modulate the regulatory effect of TIM-3 on DCs. Moreover, as mentioned, it seems that the microenvironment has a key role in the function of TIM-3 expressed on the DC surface. For example, the ratio between GAL-9 and HMGB1 in the tumor microenvironment plays an important role in determining whether TIM-3 expression on DCs can be beneficial or detrimental to the tumor.

Recent data have shown that the highest TIM-3 expression level in human PBMCs is on NK cells, particularly on all mature CD56^dim^CD16^+^ NK cells. Also, cytokines such as IL-12, IL-15, and IL-18 could induce TIM-3 upregulation on immature CD56^bright^CD16− NK cells. Furthermore, interaction with Gal-9–TIM-3 in NK cells improves NK cell–mediated cytotoxicity and IFN-γ production. Thus, TIM-3 can be considered as a marker of full maturation and function in NK cells. Paradoxically, in other reports, NK cell responses may be downregulated if NK cells encounter target cells expressing related ligands of TIM-3. For example, in advanced melanoma patients, NK cells expressing high levels of TIM-3 are functionally exhausted, and its upregulation on NK cells is associated with poor prognosis (9, 12, 20). Also, Liu Yuan et al. have shown that blockade of TIM-3–Gal-9 interaction can induce enhanced cytotoxicity and upregulates IFN-γ production in both NK cells from patients with chronic hepatitis B and NK92 cell lines. These observations strongly suggest that, although TIM-3 can be considered as a marker of NK cell activation, its overexpression may also disrupt the function of NK cells in certain situations (9, 20) (**Table 1**).

All in all, apart from the public attention that TIM-3 has gained as an inhibitory immune regulator, its role in regulating the host immune response is complicated and remains controversial in many fields such as liver cancer.

## TIM-3 and HCC

The association of tumors and TIM-3 has been considered for many years (9). The expression of TIM-3 has been found in various types of cells in TME, including on TILs, cancer cells, and endothelial cells (ECs). Upregulated expression of TIM-3 in tumor tissue samples has been demonstrated in melanoma, prostate cancer, colon cancer, bladder urothelial carcinoma, cervical cancer, gastric cancer, liver cancer, and lung cancer cells. In all of these cancers, a higher TIM-3 expression level is correlated with poor prognosis (20, 30). Moreover, it was reported that prolonged exposure to the PD-1-blocking antibody upregulates the expression of TIM-3 in therapeutic antibody-bound CD4 and CD8 T cells in the TME (59). On the other hand, TIM-3 blockade in some cancers has been shown to downregulate CTLA-4 and TIGIT expression levels and significantly decrease the development of cancer (18). Given the recent success of immune checkpoint therapy in cancer, currently, TIM-3 has received increasing attention (9). However, the exact role of TIM-3 in liver cancer is still unclear.

**Figure 2:**
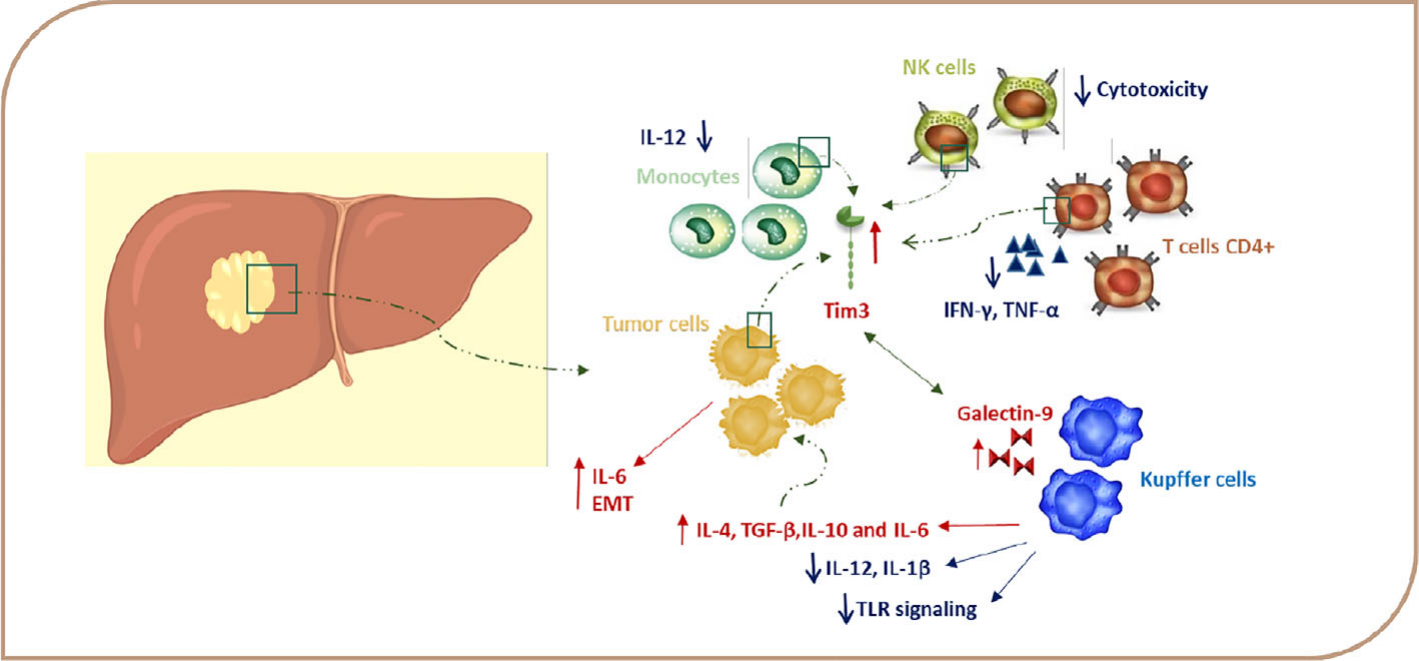
TIM-3 expression in HCC. Signaling *via* TIM-3 after binding to a ligand regulates immune responses *via* negatively regulating Th1 cell viability and secretion of cytokines, such as TNF and IFN-γ, and this, in turn, leads to immune tolerance. Gal-9–TIM-3 interaction downregulates TLR signaling, leading to a decrease in IL-12 and IL-1β production and increase of IL-4, IL-6, TGF-ß, and IL-10 production. Due to the presence of cytokines, such as IL-4, TGF-β, and IL-6 in the TME, TIM-3 expression is induced in HCC cells, and this upregulation leads to tumor growth through autosecretion of IL-6 and enhancement in the metastatic ability by promoting EMT. TIM-3–Gal-9 interaction can decrease cytotoxicity in NK cells. EMT, epithelial-mesenchymal transition; Gal-9, galectin-9; HCC, hepatocellular carcinoma; IFN, Interferon; IL, interleukin; TGF, transforming growth factor; TNF, tumor necrosis factor.

Several studies demonstrate that the expression of TIM-3 in HCC cells is induced due to the presence of cytokines, such as IL-4, TGF-β, and IL-6 in the TME (60). The expression of TIM-3 in HCC cells is accelerated tumor growth *via* autosecretion of IL-6, and this also increases the metastatic ability of HCC cells by promoting epithelial-mesenchymal transition (EMT) (**Figure 2**) (61).

On the other hand, TIM-3 expression is also induced on immune cells due to chronic stimulation and the cytokine milieu in the TME (60). Wu et al. find that TIM-3 expression is increased on CD4^+^ and CD8^+^ T cells during chronic hepatitis, which leads to decreasing plasma IFN-γ and T-bet mRNA levels in these cells (**Figure 2**). Moreover, the TIM-3 expression level is associated with the severity of the disease and is positively correlated with alanine aminotransferase (ALT), aspartate aminotransferase (AST), the international normalized ratio (INR), and total bilirubin (TB) (62). Also, accumulating evidence supports the possibility of a regulatory role for TIM-3 on TILs, leading to immunosuppression in HCC TME. Accordingly, Yan W et al. have detected increased TIM-3 expression levels on CD4^+^ T and CD8^+^ T cells from peripheral blood of HCC patients (47). Moreover, Liu Y et al. demonstrate that the expression of TIM-3 is elevated on CD4^+^ and CD8^+^ T cells infiltrating tumor tissues in comparison with those cells infiltrating the neighboring tissues (9, 47). Also, Jie Ji et al. have reported that the expression of Lnc-TIM-3 was highly upregulated in the TILs of HCC patients. This LncRNA binds to the intracellular tail of TIM-3, so it suppresses the downstream signaling pathway NFAT1 and AP-1 due to the release of Bat3 from the TIM-3 tail in CD8 T cells (63). Zhou G et al. demonstrate that T cells expressing PD-1 and TIM-3 display the lowest level of granzyme B, IFN-γ, and TNF-α and, hence, suggest that PD-1 and TIM-3 play a key role in T cell suppression in the HCC microenvironment (64). In accordance, the frequency of TIM-3 positive tumor-infiltrating T cells is correlated with a poor prognosis, and TIM-3–Gal-9 signaling promotes T cell senescence. In the HCC, Kupffer cells have the highest level of Gal-9 expression due to their response to IFN-γ derived from TILs and are co-localized with TIM-3 positive T cells (**Figure 2**). Blocking of the TIM-3–Gal-9 signaling pathway reactivates T cell–mediated antitumor immunity as confirmed by the enhanced proliferation of T cells and increased cytokine production in the HCC microenvironment. Therefore, TIM-3 might be a great potential target in HCC therapy by stimulating both innate and adaptive immune responses (9, 12, 47). The results of these studies suggest that, in HCC, it is better to use blocking antibodies against PD-1 and TIM-3 together for significant results in the immunotherapy of HCC.

Recent studies have shown that, due to the presence of enhanced TIM-3 on monocytes in chronic hepatitis B patients, this molecule plays a key role in the pathogenesis of chronic hepatitis B (65). According to these results, it is reported that the TIM-3 expression on peripheral blood monocytes is higher in patients with HCC compared to controls, and there is a negative correlation between TIM-3 expression and a patient’s survival. Wenjiang Yan et al. show that enhanced expression of TIM-3 on tumor-associated macrophages (TAMs) in hepatocellular carcinoma is triggered by tumor-derived signals, including TGF-β (11, 20, 47). Interestingly, the expression of TIM-3 is even higher in M2 monocytes, which are traditionally described as inducers of tumor progression (9, 47, 58). A TIM-3 knockdown assay in LPS/IL-4-stimulated bone marrow-derived monocytes (BMDMs) has shown that, in either TGF-β-dependent or -independent pathways, TIM-3 can induce polarization toward M2 macrophages. Recently, the important role of NF-κB in maintaining the M2 phenotype and protumoral function of TAMs has been emphasized in various cancers, including HCC. Yan W et al. show that TIM-3 mediates the activation of the NF-κB pathway in TGF-β or IL-4-stimulated macrophages. In one study, blockade of TIM-3 expression on macrophages led to the inhibition of HCC growth both *in vitro* and *in vivo*, which is probably due to the reduced production of IL-6 by macrophages (47). Also, it was reported that tumor-inﬁltrated conventional NK (cNK) and liver resident NK (LrNK) cells showed elevated TIM-3 expression in HCC patients (**Figure 2**), which, in turn, suppresses their cytokine secretion and cytotoxicity because TIM-3 disrupts the downstream Akt/mTORC1 signaling in NK cells (66). Indeed, TIM-3 is not only expressed on immune cells, but is also expressed in HCC cells and serves as an oncoprotein in these cells (9, 47). These novel data propose that TIM-3 may have other functions in addition to the inhibition of immune responses. All of these results further emphasize the important role of TIM-3 as a new player in HCC progression and a potential target in HCC immunotherapy.

## Clinical Studies on TIM-3 in HCC and Other Cancers

Different studies have shown that TIM-3 expression is correlated with HCC outcome. Hang Li et al. showed that the higher number of TIM-3^+^ TIL T cells in HCC tissue is correlated with shorter survival of patients (67). Besides this, there are positive correlations between TIM-3 expression on CD14^+^ monocytes with tumor grade and TIM-3 expression on TAMs with poor prognosis in HCC patients (47). It is also shown that TIM-3 expression in PBMCs may predict recurrence in therapeutic liver-restricted HCC patients (68). Also, studies demonstrate that polymorphism in the promoter region of the TIM-3 gene can predispose HBV-related cirrhosis and HCC (69, 70) because the level of TIM-3 expression, remarkably, is correlated with its gene polymorphisms (71). Overall, an increase in TIM-3 expression in PBMCs, TAMs, and TIL T cells in HCC indicates poor prognosis, more advanced tumor grades, shorter survival, and a higher probability of recurrence. These findings support that the use of antibodies against TIM-3 can restore responses of HCC-derived T cells to tumor antigens and improve overall survival and treatment response in HCC patients.

There are a few clinical trials about the use of antagonistic anti-TIM-3 monoclonal antibodies in cancers, especially HCC, that have been registered on ClinicalTrials.gov. MBG453 is an anti-TIM-3 mAb for which the efficacy and safety have been evaluated in first clinical trials as a single agent or in combination with PDR001 (anti-PD-1 mAb) in advanced malignancy patients (NCT02608268). It was also conducted in patients with acute myelocytic leukemia or high-risk myelodysplastic syndromes (NCT03066648) (72). TSR-022 is another anti-TIM-3 mAb for which its efficacy and safety were assessed in patients with an advanced solid tumor alone or in combination with TSR-042 (NCT02817633). There are also two other clinical trials on TSR-022 (NCT03680508, NCT03307785) (73, 74). NCT03680508 is a phase II trial that is studying the effect of TSR-022 in combination with TSR-042 (anti-PD-1 antibody) in the treatment of patients with locally advanced or metastatic liver cancer, and it has begun recruitment. Sym023 is another anti-TIM-3 mAb in phase I clinical trials in patients with metastatic cancer, solid tumor, and lymphoma (NCT03489343). There are other TIM-3 inhibitors in the phase I trial, such as BMS986258, LY3321367, SHR1702, and INCAGN2390 (61, 75). RO7121661 is a bispecific antibody targeting TIM-3 and PD-1 and has been used in patients with advanced and/or metastatic solid tumors in a phase 1 study; results are awaited in May 2023 (NCT03708328) (76). In a meta-analysis by Qin et al., a total of 3072 patients were included, and their results suggested that TIM-3 protein overexpression was relevant to poor overall survival. They also showed that TIM-3 was connected with tumor grade, PD-1 expression, and lymph node metastasis. Finally, they concluded that TIM-3 has the potential to be a prognostic marker and a valuable therapeutic target in solid tumors (77).

## Metabolic Situation in HCC and the Role of TIM-3

The metabolism, phenotype, and function of immune cells are affected by the presence and level of the nutrition molecules in the TME, such as fatty acids and glucose, which is the concept of “immunometabolism.” Glucose deficiency and lactic acid abundance in the TME limit the activity of CTLs, and enhancement of fatty acid oxidation (FAO) blocks the cytotoxicity of CTLs in tumors, whereas Treg cells use FAO instead of glycolysis, so these cells survive in glucose-deficient and hypoxic environments and suppress the tumor-killing function of CTLs (1, 2, 5). Elevated aerobic glycolysis, gluconeogenesis, and β-oxidation of fatty acids, along with reduced TCA cycle activity, are major metabolic alterations in liver cancer. Thus, similar to effector T cells, liver cancer tumor cells predominantly use glycolysis even in the presence of sufficient oxygen pressure. On the other hand, lactate is released into the extracellular environment by monocarboxylate carriers (MCT) as a result of increased glycolysis activity, and accordingly, an overexpression of MCT4 has been reported in HCC samples (78). A high rate of lactic acid production by liver cancer cells through glycolysis is another nonnegligible issue when considering the metabolic reprogramming of liver cancer–associated immune cells. Lactate can increase the number of MDSCs, thereby influencing the other cell types and also inhibiting the function of NK cells. The immunosuppressive functions of MDSC are also regulated by other metabolites, such as fatty acids (78). In liver cancer, an elevated β-oxidation fatty acid compensates energy deficiency and also reduces tumor dependence on glucose (5). However, deregulation of mitochondrial β-oxidation and many enzymes involved in fatty acid oxidation in HCC tumor cells has been reported (78, 79). Some convincing evidence shows that the oxidative metabolism of MDSCs is increased by the accumulation of lipids, which also, in turn, activates their immunosuppressive mechanisms (78). M2 polarized macrophages need oxidative phosphorylation (OXPHOS) and FAO to obtain the required energy and exert their protumor function in various conditions, such as liver cancer (5, 78). Accordingly, patients with obesity, diabetes, and hepatic steatosis demonstrate an increased risk of HCC pathogenesis as the liver plays a key role in the metabolism of lipids and lipoproteins (78, 80). PD-1 signaling switches T cell metabolism from glycolysis to FAO by reducing AKT activation and, thus, suppressing mTOR activity (81, 82). Also, PD-1 signals inhibit the expression of key metabolic regulator peroxisome proliferator-activated receptor gamma co-activator 1-alpha (PGC1α), the overexpression of which in tumor antigen–specific CD8^+^ T cells could enhance antitumor responses by promoting mitochondrial biogenesis (83). However, the exact mechanisms through which TIM-3 alters T cell metabolic program remains to be elucidated. TIM-3–Gal9 interaction targets the main metabolism-associated signaling pathways, such as PI3K/Akt/mTOR, and is highly expressed on dysfunctional or exhausted T cells in TILs in various types of cancers. TIM-3–mediated effects may alter the metabolism of effector T cells in a way similar to PD-1 (8).

Also, increasing evidence has shown that TIM-3 expressed on tumor cells and tumor-associated immune cells have pleiotropic functions that affect several immunologic and biologic aspects of different types of cells (30). For example, in AML cell lines (HL-60), it is shown that TIM-3, through moderate activation of the mTOR, can activate HIF-1α, which upregulates glycolysis and expression/secretion of the pro-angiogenic vascular endothelial growth factor (VEGF) (15, 31, 84). Because TIM-3 is an important immune checkpoint in HCC, knowing the immunometabolism pathways that are affected by TIM-3 is an interesting subject that should be studied in the future. These studies may help us to discover better immunotherapy targets in HCC.

## Concluding Remarks

Increasing the percentage of patients who may respond to anticancer therapies is one of the main challenges in immunotherapy. An accumulating body of evidence confirms the importance of TIM-3 as an immune checkpoint in various human cancers, including HCC. Although its roles as an immune checkpoint in immune and tumor cells have gained much attention, the role of TIM-3 in metabolic reprogramming of immune and cancer cells in the context of HCC has not yet been thoroughly investigated. Based on the importance of HCC as a severe problem in global health and also because of the paucity of information about the role of TIM-3 as a metabolic checkpoint, the investigation and determination of the expression pattern of TIM-3 and its ligands in the HCC TME are of importance. The expression of TIM-3 and its ligands on cancer and immune cells have been shown in HCC TME. Because interaction of TIM-3 to its ligands makes an immunosuppressive situation in TME, blocking the TIM-3 or its ligands can be a promising target for immunotherapy in HCC. In addition, determining the role of TIM-3–Gal-9 interaction versus TIM-3 inhibition on TIL, M2, MDSC, and tumor cell metabolic reprogramming in order to define the crosstalk between metabolic reprogramming of immune cells and cancer cells in this malignancy is highly recommended.

## Author Contributions

MG and AB conceptualized the manuscript. MG generated the figures. All authors contributed to the article and approved the submitted version.

## Conflict of Interest

The authors declare that the research was conducted in the absence of any commercial or financial relationships that could be construed as a potential conflict of interest.
